# Tectonic and climatic impacts on the biota within the Red River Fault, evidence from phylogeography of *Cycas dolichophylla* (Cycadaceae)

**DOI:** 10.1038/srep33540

**Published:** 2016-09-15

**Authors:** Ying Zheng, Jian Liu, Xun Gong

**Affiliations:** 1 Key Laboratory for Plant Diversity and Biogeography of East Asia, Kunming Institute of Botany, Chinese Academy of Sciences, Kunming 650201, Yunnan, China; 2 University of Chinese Academy of Sciences, Beijing 100049, China

## Abstract

Dramatic crustal deformation and river incision in Southwest China induced by the Indo-Asian collision have long been argued to contribute to the complicated landscapes, heterogeneous environment and abundant biodiversity in this region. However, biological impacts in promoting intraspecific phylogeographical subdivision and divergence along the Red River Fault zone (RRF) remain poorly understood. To investigate the possible biological effects of tectonic movements and environment variations within the RRF, the phylogeography of *Cycas dolichophylla*-an endemic but widely distributed *Cycas* in Southwest China and North Vietnam along the RRF were carried out based on four chloroplast DNA intergenic spacers (cpDNA), three nuclear DNA sequences (nDNA) and 16 simple sequence repeat variations (SSR). Two different phylogeographical patterns were detected: a Southwest-Northeast break across the RRF disclosed by chlorotypes and a China-Vietnam separation revealed by SSR. A Bayesian skyline plot from cpDNA data demonstrated a historical increasing, but a recent declining, dynamic in population size during the Pleistocene. Consequently, we infer it is the local environmental variation during Cenozoic that contributed to the complex landscape and microclimate mosaics, facilitating speciation and divergence of *C. dolichophylla*. Subsequently, the Quaternary climatic fluctuations coupled with human activities profoundly influenced the genetic structure and demographic history of this species.

In recent years, accumulating evidence from tectonics, paleontology and climate simulations increasingly supported a view that the main body of the Tibetan Plateau (TP) reached its current height (4–5 km) since the mid-Eocene^1^,^2^,^3^,^4^, that is, the uplift of TP and the evolution of Asian monsoon systems contribute little to the speciation and diversification in Southwest China^1^,^2^,^3^,^4^,^5^. Contrarily, it might be the local ecological habitat diversity that resulted in the numerous young plant and animal clades endemic to such a biodiversity hotspot^1^,^6^. The Red River Fault zone (RRF), defined by Indochina in the southwestern and South China in the northeastern, is proposed to be the reactive product of India-Asia collision^7^. It has been argued experiencing left-lateral strike-slip in the first 30–16 Ma^8^ of collision, yielding at least 15 km of displacement^9^ and presently right-lateral motion since the late Neogene (8–7 Ma)^10^ with lower slip rate^11^,^12^. However, the emergence of extant *Cycas* worldwide is supposed to be not much older than ~8 Ma^13^, the displacement generated by strike-slip stress may have had minimal influence on the speciation of this lineage. Reconstruction of the palaeo-Red River indicated a river incision of nearly 1400 m^14^, shaping a bulk of the landscape mosaic, which might serve as buffers or shelters for species to survive. Moreover, climatic oscillations during the Quaternary have exerted significant influences on casting genetic diversity and historical dynamics of animals and plants in Southeast China by altering species distributions^15^. Thus, it might be the regional variation of climate, environment as well as microclimate establishment, that acted as major thrust for modifying and influencing genetic discontinuities, speciation, and diversification of organisms^16^,^17^,^18^,^19^. However, details of spatiotemporal evolution and biological effects of the RRF in promoting intraspecific phylogeographical subdivision and biotic diversification still remains poorly understood.

*Cycas dolichophylla* K. D. Hill, T. H. Nguyen & K. L. Phan is an ideal species for inferring the potential biological/biogeographical model within the RRF. On one hand, the distribution of this organism is restricted to the Red River drainage region (Fig. 1) with poor water dispersal capability (no spongy layer inside its seed)^20^. On the other hand, it belongs to the oldest and most primitive groups of the extant seed plants, which originated before the mid-Permian, reached diversity peak in the Jurassic-Cretaceous with ranges far across the Northeast China^21^, and underwent massive historical extinction and a recent synchronous global re-diversification during the late Miocene^13^. Hence, considering the historical geological and paleo-climatic evidence along with the present distribution pattern of *C. dolichophylla* between China and Vietnam along the RRF, we tried to test if the temporal divergence of intraspecific phylogroups would be connected with effects of geological and climatological events. Alternatively, it also remains to be clarified whether the geological data reviewed above accords with lineage divergence time derived from molecular dating of *C. dolichophylla*.

Here, a phylogeographical study of *C. dolichophylla* was conducted by tracing variations from four chloroplast intergenic spacers (cpDNA), three nuclear sequences (nDNA) and 16 simple sequence repeats (SSR) in 13 populations across its entire range with an attempt to test the following hypotheses: (i) local environmental variation in its habitats influenced the lineage divergence of *C. dolichophylla*; (ii) climate variations, especially the glacial-interglacial cycles during the Quaternary re-sculptured this species’ genetic structure and accelerated its diversification.

## Results

### Phylogeographical patterns

**Figure 1:**
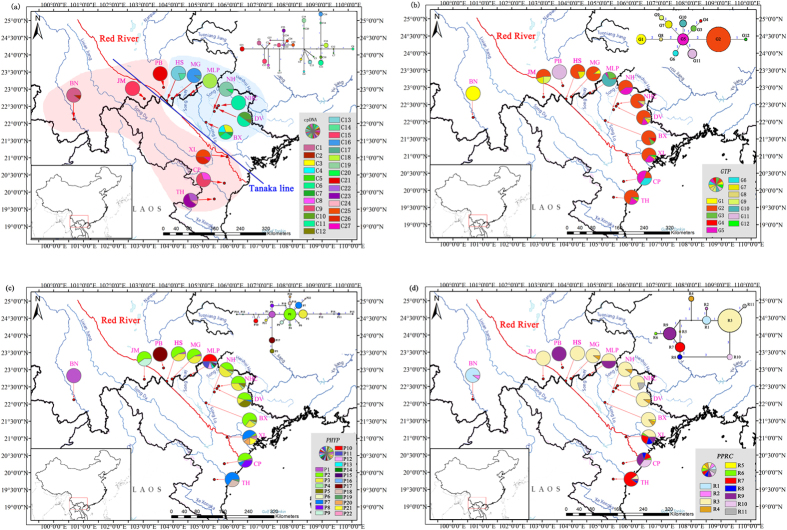
Sampling sites and geographical distributions of chlorotypes (**a**) and nuclotypes ((**b**) *GTP*; (**c**) *PHYP*; (**d**) *PPRC*) identified in the 13 populations of *C. dolichophylla* along the RRF, with networks of each haplotype displayed on the upper right corner. The sizes of the circles in the networks are proportional to the observed frequencies of the haplotypes and numbers above the lines mean the mutational steps. The black dots are the missing haplotypes. The red curve represents the Red River, separating this species into two lineages: the pink shadow area comprises populations from southwestern clade and the light blue area occupied by northeastern clade. The blue straight line in Fig. 1a stands for the modified ‘Tanaka line’. Maps were drawn using the software ArcGIS version 10.2 (http://desktop.arcgis.com) and modified using Photoshop (Adobe Corporation, California, America).

**Figure 2:**
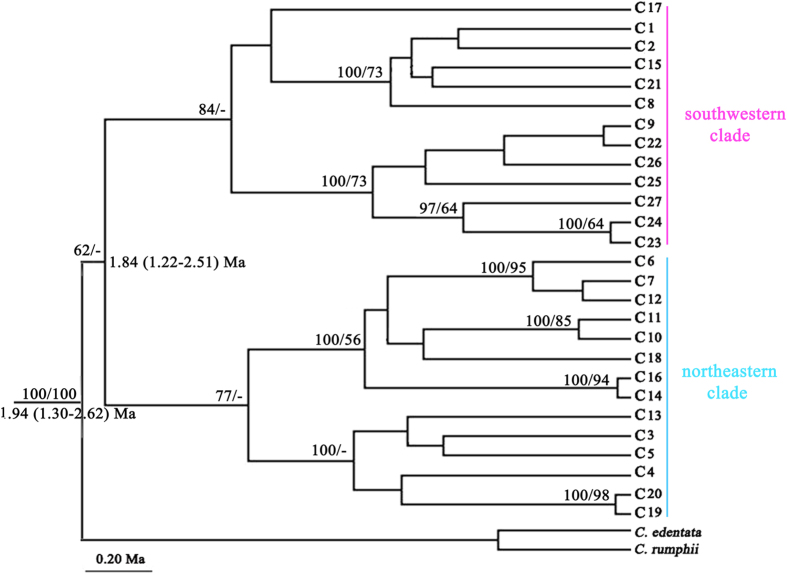
BEAST-generated maximum clade credibility tree of chlorotypes from 13 *C. dolichophylla* populations, with *C. edentata* and *C. rumphii* set as outgroups. The topologies derived from Bayesian Inference and Maximum Parsimony accord with BEAST trees, except for minor incongruences at nodes with low confidence level. Numbers above the branches indicate posterior probabilities (left) and bootstrap values (right) from the Maximum Parsimony principle (>50 are shown). Numbers below branches denote the divergence times with 95% HPD.

After alignment, a conjoined cpDNA matrix was acquired with 3687 bp in length, identifying 72 polymorphism loci in 27 haplotypes (Table 1). Among these chlorotypes, 11 (C1, 2, 8, 9, 15, 22–27) occurred in the southwest populations of the RRF (BN, CP, JM, XL and TH), whereas the other 16 were endemic to the northeastern populations of the RRF (BX, DV, HS, MG, MLP, NH, NHC and PB) (Fig. 1a). The maximum clade credibility tree generated by BEAST uncovered the crown age of *C. dolichophylla* to be 1.94 Ma (95% HPD: 1.30–2.62 Ma) and retrieved the ‘northeastern’ and ‘southwestern’ chlorotypes as reciprocally monophyletic lineages, with coalescence time dated to about 1.84 Ma (95% HPD: 1.22–2.51 Ma) (Fig. 2). AMOVA revealed that most variations occurred among populations (83.68%) with only 16.32% existed within populations, which in accordance with results of northeast and southwest populations (Table 2). The high value of *F*st (*F*st > 0.25) suggested significant genetic differentiation among populations based on Wright’s^22^ assumption. However, gene flow detected by cpDNA data implied that genetic exchanges between chlorotypes were quite low (*N*m < 1) (Table 2).

The aligned length of three nuclear genes, *GTP*, *PHYP* and *PPRC,* were 573–574 bp, 940–946 bp as well as 720 bp, respectively. For *GTP*, 14 polymorphism loci were identified with 12 haplotypes confirmed (Table 1). Haplotype 1, 7, 8, 9 (G1, 7–9) clustered together, while the others formed another lineage with the divergence time closing to 0.64 Ma (95% HPD: 0.25–1.09 Ma) (Fig. 1b; Supplementary Fig. S1a). G2 was the most common haplotype, occurring in 11 of the 13 populations, with its geographic distribution range covering both southwestern and northeastern of the RRF. Nevertheless, although it was not a widely shared haplotype, G5 occupied an interior (ancestral) rather than tip (derived) position in the network with the derived haplotypes showed a satellite-like distribution (Fig. 1b). All of the individuals sampling from PB were restricted to a private haplotype (G11), whereas the BN population was fixed by G1 with one MG sample included (MG-9). As for *PHYP*, 22 haplotypes were defined based on 26 polymorphism loci (Table 1). These haplotypes were further grouped together in two clades, namely, P5 and P17 formed one clade and the other 20 haplotypes shaped another one where four mutational steps were taken for this separation (Fig. 1c). The timing of most recent common ancestors of these two lineages was estimated to 0.58 Ma (95% HPD: 0.28–0.92 Ma) (Supplementary Fig. S1b). Similar to the results of *GTP*, population PB still occupied a private haplotype (P17) and BN was restricted to P1 although this haplotype was shared by CP, HS, MG and XL (Table 1). Interestingly, the population of MLP defined seven distinct haplotypes (P10–16) by a unique 34 bp or 68 bp deletion accompanied by several mutations. The most widely shared haplotype, P2, was in the interior position of the network with nine populations embraced and the other haplotypes formed a star-like structure including two small ‘loops’ (Fig. 1c). Eleven haplotypes were identified on the base of 11 polymorphism loci of *PPRC*. Two lineages were also confirmed by the maximum clade credibility tree with divergence time approaching to 0.53 Ma (95% HPD: 0.21–0.87 Ma) (Supplementary Fig. S1c). However, relationships inferred from Network remained ambiguous because of the presence of multiple closed ‘loops’ (Fig. 1d). Apart from BN, CP and PB, haplotype 3 (R3) swept all of the remaining populations occurring in either southwest or northeast of the RRF. No matter in MP-derived strict consensus trees or the maximum clade credibility trees inferred by BEAST, all the combined cpDNA and three nDNA sequences suggested the monophyly of *C. dolichophylla* with fine support (MP bootstrap for cpDNA: 100%; *GTP*: 86%; *PHYP*: 86%; *PPRC*: 86%) (Fig. 2; Supplementary Fig. S1a–c).

As for SSR data, a total of 217 alleles were identified by 16 microsatellite loci in 255 individuals from 13 populations (Supplementary Tables S1 and S2). Results of Bayesian clustering inference (Supplementary Fig. S1e,f) indicated that the most likely number of clusters was 2 for all the *C. dolichophylla* populations when the ΔK statistics were applied. When considering the second fittest number of grouping, which suggested as K = 3, it was almost similar to the case in K = 2 with only minor subdivision. Additionally, all the structure analysis derived from UPGMA and PCo (Supplementary Fig. S1d,h) supported for two grouping division. The BARRIER analysis (Supplementary Fig. S1g) showed that there was only one major genetic boundary with a 58% mean bootstrap value, separating BN from the remnant twelve populations. Therefore, two lineage clusters corresponding to cluster I and cluster II in UPGMA were applied in this study.

The AMOVA analysis (Table 2) reflected that there were nearly parallel contributions on molecular variations both among and within populations derived from *PHYP* (50.90% vs 49.10%), *GTP* (55.08% vs 44.92%) and *PPRC* (59.24% vs 40.76%). However, 74.3% of genetic variations within populations and 25.7% among populations were detected from SSR data. The fixation indexes calculated by these three nuclear genes as well as SSRs were all greater than threshold 0.25, indicating significant genetic variation between populations. The gene flows inferred from nuclear genes were lower than the critical value 1 (Table 2). However, results of analysis of gene flows between each pair of the 13 populations in *C. dolichophylla* based on SSR data (Supplementary Table S3) were disparate with cpDNA and nDNA, which exhibited that XL and BX exchanged genetic materials more frequently with other populations no matter from South Yunnan or Vietnam than others. Moreover, the highest level of gene flow was detected between DV and NH whose geographic distance was considered to be the closest among these populations.

### Population genetic diversity and structure

For the cpDNA data, the average within-population diversity (*H*_S_ = 0.320) was much lower than the total diversity (*H*_T_ = 0.994), and both *N*_ST_ (0.834) and *G*_ST_ (0.678) were high (Table 3). U-test showed that *N*_ST_ was significantly larger than *G*_ST_, indicating a phylogeographical structure of chlorotype distribution. Moreover, the genetic diversity detected in northeast and southwest populations were similar (*H*_S_ = 0.321 vs. 0.319; *H*_T_ = 0.976 vs. 1.000), which was different from estimating of population subdivision (*N*_ST_ = 0.740 vs. 0.823; *G*_ST_ = 0.672 vs. 0.681), that was, significant phylogeographical structure was detected in southwest (P = 0.018) but not in northeast (P = 0.051). In accordance with results of phylogeographical structure inference, Mantel test uncovered a significant pattern of IBD for cpDNA at the range-wide scale (r = 0.281, P < 0.05) (Supplementary Fig. S2) and in southwest, but not the case in northeast.

The genetic diversities (Table 3) were much higher in *PHYP (H*_S_ = 0.524; *H*_T_ = 0.869) than in *GTP (H*_S_ = 0.387; *H*_T_ = 0.719) or *PPRC (H*_S_ = 0.291; *H*_T_ = 0.670). Consequently, both the *N*_ST_ and *G*_ST_ estimated by *PHYP* were lower than the other two genes. However, no significant difference between *N*_ST_ and *G*_ST_ was detected on these three gene fragments. In other words, no significant effect of IBD was detected, as the correlation between genetic distance and geographic distance was not significant (P > 0.05).

### Historical demography based on DNA sequence and SSR variation

The cpDNA sequence mismatch analysis results (Supplementary Fig. S3a) for all the *C. dolichophylla* populations as well as northeastern and southwestern groups displayed a multimodal distribution pattern with non-significant SSD statistic and *H*_Rag_ value under a sudden demographic expansion model, except for the significant SSD value detected at the whole chlorotypes level. Similarly, positive values of Fu and Li’s D*, Fu and Li’s F*, Fu’s Fs rejected the sudden expansion model (Supplementary Table S4). For nDNA, multimodal or bimodal distributions were detected with the appearance of non-significant SSD and *H*_Rag_ (Supplementary Fig. S3b–d). The results of neutrality test and Fu’s Fs for *GTP* and *PPRC* were similar, with positive but not significant values acquired. Nevertheless, the case for *PHYP* was discrepant: all the SSD statistic and *H*_Rag_ value were non-significant, along with negative results of neutrality test and Fu’s Fs (except for Fu and Li’s D*) (Supplementary Table S4).

**Figure 3:**
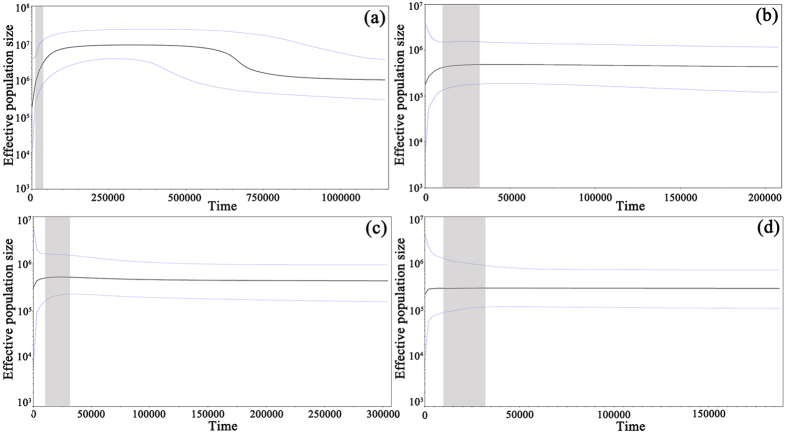
Bayesian skyline plots based on cpDNA (**a**) and nDNA ((**b**) *GTP*; (**c**) *PHYP*; (**d**) *PPRC*) for the effective population size fluctuation throughout time. The gray areas indicate the last stage of the last glacial period (32–12 ka).

Historical demography inferred by Bayesian skyline plot based on a jointed cpDNA matrix uncovered two dynamic events: during about 0.57–0.70 Ma, *C. dolichophylla* experienced population expansion (Fig. 3a). However, it began to contract in effective population size at approximately 0.10 Ma with a rapid decline rate detected during about 70 Ka. Population demographic histories deduced from nDNA revealed that *C. dolichophylla* have experienced population size contraction mainly during the late Pleistocene to Holocene (Fig. 3b–d).

For both the Sign and Wilcoxon tests (Supplementary Table S5), no significant heterozygosity excess was detected under the S.M.M assumption. However, on the postulation of a more conservative and powerful model (T.P.M), Sign test revealed significant excess of heterozygosity in PB. As for the Wilcoxon test, it seemed to indicate that BN and CP have experienced significant heterozygosity excess, while NHC and PB suffered the most significant excess of heterozygosity, all of which implied a departure from mutation-drift equilibrium^23^. Moreover, a normal L-shaped mode-shift distribution was forecasted in each of the population. All the G-W indices did not exceed the threshold level of 0.68 (Supplementary Table S5).

## Discussion

A major finding of the present study was that *C. dolichophylla* comprised two geographically distinct lineages as inferred from the chlorotype network analysis, namely a southwestern clade distributed to the southwest of the RRF (including PB) and a northeastern clade occupying the northeast region (Fig. 1a). This genealogical split was geographically largely consistent with one phytogeographical boundary, ‘Tanaka Line (TKL)’^24^, except for two southwestern-clade chlorotypes, C17 and C21, that occurred in the northeast of RRF. These two chlorotypes escaping from southwest lineage was best explained by the local adaptation to novel selective pressures during the species’ range expansion into geographically and ecologically different environments. However, the missing of ten interior haplotypes was mainly due to the species’ long evolutionary history during historical climate oscillations, geological as well as recent human activities^25^,^26^.

The network of nuclear haplotypes, no matter in *GTP*, *PHYP* or *PPRC*, all failed to register a prominent genealogical break across the RRF although several exclusive haplotypes were discovered in southwestern and northeastern clades, respectively (Fig. 1b,c). Phylogenetic relationships of nucleotypes inferred from BEAST trees remained confusion (Supplementary Fig. S1a–c). Such phylogenetic ambiguity could be explained by the widely shared nucleotypes between southwestern and northeastern populations (e.g. G2, P2 and R3), which may to the retention of ancestral polymorphisms^27^. Interestingly, the results of SSR data (UPGMA and Bayesian clustering inference) revealed another distinct scene (Supplementary Fig. S1d,f): *C. dolichophylla* was split into two groups divided by national boundary, a Chinese group with populations from BN, JM, MLP and PB and a Vietnam group with the populations from northern Vietnam, as well as two Chinese populations, HS and MG. The explanation for two Chinese populations nested in Vietnam lineage might be the closer geographical distance to Vietnam, accompanied with the influence of southern drained Red River system as well as the frequent human activities, such as commercial trades and the introduction of exotic populations. The observation of much stronger phylogeographical division across the RRF in cpDNA as opposed to nDNA and SSR is associated with the different inheritance modes and evolutionary rates between nuclear and organelle genomes^28^.

The discovery of two distinct groups with cpDNA data was not unexpected, given the complex topography and heterogeneous environmental conditions in Southwest China, especially along the RRF. However, for a newly radiated species, the local habitat differences in the immense region with varying altitudes, soils, slopes as well as ecological selection from pollinators and seed transporters, seemed more promising factors to explain the diversification of *C. dolichophylla* than geological uplift. On one hand, the re-diversification of *Cycas* mainly happened in the late Miocene^13^,^29^ and the divergent time derived from chlorotypes of *C. dolichophylla* was during the early Pleistocene (1.94 Ma). On the other hand, most of the tectonic process in Southwest China happened during or even before the Miocene, such as uplift, crustal thickening, strike-slip and river incision^1^,^2^,^3^,^4^,^11^. Therefore, we proposed that it is the heterogeneous topography along the Red River region that acted as physical barriers to restrict gene flow between cycads, with global climate changes^30^ resulted in the diversification of *Cycas* species in this region. Subsequently, the local environmental variation contributed to the formation of microclimate pockets^31^, providing suitable and stable environment for species to survive through hostile climate fluctuation and accumulate genetic variations that gave rise to the speciation of *C. dolichophylla*, as well as the subsequent differentiation of the Southeastern and Northwestern subclades^32^. However, the fluctuation of Quaternary climate also made particular impacts on the population dynamics of this newly formed species, which was evident from our BSP inferences and neutrality tests (Fig. 3; Supplementary Table S4).

During the largest Naynayxungla Glaciation (0.72–0.50 Ma) when valley glaciers dominated^33^, it was difficult for this cliff species to flourish, but underwent range contraction, limiting intraspecific gene flows and further diversification of nDNA lineages. The subsequent interglacial period provided opportunity for *C. dolichophylla* to undergo spatial expansion and population growth. Nonetheless, with the persistence of cold conditions until the late Ionian Stage (0.3–0.13 Ma) together with the onset of Riss glacial cycle, the enlarged *C. dolichophylla* populations began to shrink. Moreover, during the last stage of the last glacial period (32–12 ka), cold and arid climatic conditions established, accompanied by a strengthened Asian winter monsoon dominating over continental Southeast Asia^34^. Being adapted to warm and moist conditions, such abnormal climate variations restricted *C. dolichophylla* to scattered habitats and thus hindered gene flow between populations. Although the Bottleneck analysis based on SSR data failed to register any recent bottleneck event on this species, the small G-W indices implied a historical bottleneck, which coincided with the demography history (Supplementary Table S5). Concurrently, genetic drift probably played a key role in differentiation, regarding the small historical population size of *C. dolichophylla* estimated by LDNe (Supplementary Table S6).

In conclusion, our results together with evidence from previous researches jointly support the perspective that it is the climate changes and environmental heterogeneity (pockets of microclimate) that gradually cast the evolutionary history of *C. dolichophylla*. That is, tectonic activities, river incision as well as the reinforcement of Asian monsoon systems didn’t work directly on speciation, but local habitat differences coupled with ecological selection from pollinators and seed transporters gave birth to the speciation of *C. dolichophylla.* Whereas the alternation of glacial and interglacial periods from the mid-Pleistocene onwards played critical roles in shaping this species’ population structures and demographic history.

## Methods

### Population sampling

A total of 255 foliage samples from 13 populations of *C. dolichophylla* in RRF were collected, which covered almost the entire range of this species (Fig. 1; Supplementary Table S7). Each population was represented by 11–28 individuals. Fresh leaves were dried in silica gel immediately after collection for DNA extraction. Ten individuals per population were randomly selected for chloroplast and nuclear DNA sequencing, with *C. edentata* and *C. rumphii* being employed as outgroups. All of the 255 individuals were used for the microsatellite study.

### DNA extraction, amplification and sequencing

Genomic DNA was extracted from the silica-dried leaves by modified CTAB method^35^. Four cpDNA intergenic spacers, *atp*H-*atp*I^36^, *psb*M-*trn*D^36^, *trn*L-*trn*F^37^ and *trn*S-*trn*G^36^, as well as three single-copy nDNA genes, *GTP*-binding protein Era (*GTP*)^38^, phytochrome P (*PHYP*) and hypothetical protein (*PPRC*) (Chiang, Y. C. unpublished data), were selected for subsequent PCR amplifications analysis. Microsatellite markers were selected from recently developed 65 nuclear microsatellites in *Cycas*^39^,^40^,^41^,^42^,^43^,^44^,^45^, in which 16 polymorphic microsatellite loci were chosen for genotyping. All the sequencing processes were operated by ABI 3730 automated sequencers at Shanghai Majorbio Bio-pharm Technology Co., Ltd. Sequences used in this study are available from GenBank with the accession numbers: KT824880-KT824883, KT824886-KT824889, KT901304-KT901411.

### Data analysis of DNA sequences

Prior to conducting all analyses, congruence test was generated by PAUP* 4.0 b10^46^ for the four cpDNA sequences. Although the result suggested indistinctive degree of homogeneity between those fragments (P < 0.5), a combined cpDNA matrix was still applied for the following analyses under previous suggestions^47^, whereas the three nDNA sequences were analysed separately. Genetic diversity indices were calculated by DnaSP, version 5.0^48^. Permut v.1.0 was used to calculate the mean genetic diversity within populations (*H*s), total genetic diversity for species (*H*_T_) and coefficient of population differentiation (*G*_ST_, *N*_ST_)^49^. Analysis of molecular variance (AMOVA)^50^ was conducted to partition genetic variation among and within populations. All of those analyses were performed with Arlequin version 3.1^51^. To uncover the reticulate evolutionary relationships between haplotypes, networks of chlorotypes and nuclotypes were constructed on Network, version 4.2.0.1^52^. Large fragment of indels were treated as a single mutation. Phylogenetic relationships among haplotypes were inferred based on Maximum Parsimony (MP) and Bayesian Inference (BI) methods with *C. edentata* and *C. rumphii* served as outgroups. For the MP trees, multi-trees heuristic search and tree bisection-reconnection branch swapping were adopted with 1000 repeats bootstrap analysis performing on PAUP. For BI tree search, the Markov chain Monte Carlo (MCMC) runs started from independent random trees and extended for 1.0 × 10^7^ generations, were repeated twice with trees sampled every 1000 generations implemented in MrBayes 3.1.2^53^.

Divergence times among haplotypes were estimated under a Bayesian framework in BEAST 1.6.1^54^ with HKY + G and HKY applied as the most suitable nucleotide models for joint cpDNA and three nDNA fragments, respectively. Since there was no well-documented evolutionary rate available for *Cycas*, previously estimated rates for seed plants, 1.01 × 10^−9^ and 5.1–7.1 × 10^−9^ substitutions per site per year for synonymous sites (s/s/y) for cpDNA and nDNA, respectively, were adopted in this analysis^55^. MCMC chains were run for 1.0 × 10^7^ generations, sampling once every 1000 generations. All the above parameters were performed by BEAUti 1.6.1 in the BEAST package. TreeAnnotator 1.6.1 was used to create a maximum clade credibility tree with the first 10% trees discarded as burn-in^56^. Pairwise mismatch distribution and neutrality tests^57^,^58^,^59^ were operated in DnaSP. Historical demography of *C. dolichophylla* was inferred from Bayesian skyline plots, which were generated by BEAST. Maps were drawn using the software ArcGIS version 10.2 (http://desktop.arcgis.com).

### Data analysis of SSR data

The indices of genetic diversity and genetic differentiation were calculated using GenAlEx version 6.4.1^60^. Gene flow (*N*m) between populations were calculated with formula *N*m = (1-*F*_ST_)/4*F*_ST_^61^, with the *F*_ST_ acquired from Arlequin. AMOVA analysis was performed on Arlequin. Isolation by distance (IBD) was tested by performing Mantel tests in GenAlEx using a correlation of geographic distance with genetic distance for all pairs of populations. To infer genetic structures of sampled populations and individuals, unweighted pair group mean analysis (UPGMA) were carried out on TEPGA, version 1.3^62^, with 5000 of permutations. Meanwhile, an individual-based principal coordinate analysis (PC_O_) was visualized by the program MVSP, version 3.12^63^. A model-based Bayesian clustering method was applied to assign individuals from 13 populations using the STRUCTURE version 2.3^64^. Twenty independent runs were performed for each set, with grouping number (K) ranging from 1 to 20. For each run, a burn-in of 1 × 10^5^ iterations and 1 × 10^5^ subsequent MCMC steps were performed. In addition, the second-order rate of change of the natural logarithm probability of the data with respect to the number of clusters (ΔK) was evaluated to deduce the best fit number of grouping with the online tool STRUCTURE HARVESTER V.0.6.93^65^. Barrier boundary analysis was carried out to test whether there were genetic borderlines existed between populations based on genetic distance matrices performing on BARRIER version 2.2^66^.

To detect recent bottlenecks caused by reduction in effective population size, the observed gene diversity was compared with the equilibrium gene diversity given the observed number of alleles implementing in BOTTLENECK version 1.2.02^67^. The Garza-Williamson index (G-W)^68^ was also estimated for testing early shrink in population size using Arlequin software. Effective population sizes (*N*_E_) with the linkage disequilibrium (LD) method implemented in LDNe^69^ at three levels of lowest allele frequency (0.01, 0.02, 0.05) for a 95% confidence interval were calculated for conservation strategic design.

## Additional Information

**How to cite this article**: Zheng, Y. *et al.* Tectonic and climatic impacts on the biota within the Red River Fault, evidence from phylogeography of *Cycas dolichophylla* (Cycadaceae). *Sci. Rep.* **6**, 33540; doi: 10.1038/srep33540 (2016).

## Supplementary Material

Supplementary Information

## Figures and Tables

**Table 1:** Composition of haplotypes, haplotype diversity (*H*d) and nucleotide diversity (*P*i) inferred from combined cpDNA sequences and nDNA sequences of *C. dolichophylla* investigated in this study.

Code	cpDNA			*GTP*			*PHYP*			*PPRC*		
	Haplotypes (No.)	*H*d	*P*i × 10^3^	Haplotypes (No.)	*H*d	*P*i × 10^3^	Haplotypes (No.)	*H*d	*P*i × 10^3^	Haplotypes (No.)	*H*d	*P*i × 10^3^
BN	C1(9)C2(1)	0.200	0.160	G1(20)	0.000	0.000	P1(20)	0.000	0.000	R1(18)R2(2)	0.189	0.260
BX	C3(3)C4(2)C5(1)C6(3)C7(1)	0.844	1.900	G2(16)G3(2)G4(2)	0.358	2.090	P2(13)P3(5)P4(1)P5(1)	0.537	1.370	R3(17)R4(2) R5(1)	0.279	1.550
CP	C8(3) C9(7)	0.467	1.140	G2(4)G5(9)G6(7)	0.668	2.010	P1(1)P2(9) P6(1)P7(2)P8(7)	0.695	1.880	R6(1)R7(3)R8(2)R9(7)R10(7)	0.758	3.560
DV	C10(4)C11(5) C12(1)	0.644	0.420	G2(14)G5(3)G7(2) G8(1)	0.500	2.550	P2(9)P3(3)P5(7)P7(1)	0.684	4.440	R3(18)R4(2)	0.189	1.050
HS	C13(8) C14(2)	0.356	1.260	G2(16) G7(4)	0.337	2.350	P1(1)P2(11)P3(8)	0.563	0.750	R3(20)	0.000	0.000
JM	C15(10)	0.000	0.000	G2(11)G7(4)G9(5)	0.626	4.320	P2(12) P9(8)	0.505	0.530	R3(20)	0.000	0.000
MG	C16(9) C17(1)	0.200	0.380	G1(2)G2(18)	0.189	1.980	P1(1)P2(11)P3(8)	0.563	0.750	R3(18)R4(2)	0.189	1.050
MLP	C18(10)	0.000	0.000	G2(1)G3(5)G5(1)G10(13)	0.537	1.870	P10(10)P11(4)P12(2)P13(1)P14(1)P15(1)P16(1)	0.663	2.380	R3(10)R9(10)	0.526	2.920
NH	C19(9) C20(1)	0.000	0.000	G2(12)G5(8)	0.505	1.760	P2(8)P3(10)P4(1)P5(1)	0.616	1.510	R3(18)R4(2)	0.189	1.050
NHC	C11(10)	0.000	0.000	G2(17)G5(1) G8(2)	0.279	1.600	P2(10)P3(7)P4(1)P5(2)	0.647	2.210	R3(16)R11(4)	0.337	0.470
PB	C21(10)	0.000	0.000	G11(20)	0.000	0.000	P17(20)	0.000	0.000	R920)	0.000	0.000
TH	C22(3)C23(6) C24(1)	0.467	0.250	G2(14)G5(4) G7(1)G12(1)	0.489	2.070	P7(13)P18(6)P19(1)	0.511	1.080	R3(2)R7(16)R8(1) R9(1)	0.363	1.560
XL	C25(5)C26(4) C27(1)	0.644	0.670	G2(13)G5(5) G7(1)G12(1)	0.537	2.240	P1(1)P2(1)P7(9)P18(1)P20(3)P21(4)P22(1)	0.763	2.600	R3(8)R6(1)R7(5)R8(3)R9(3)	0.768	4.020
Total		0.940	2.510		0.677	3.840		0.809	2.900		0.642	3.160

*H*d: haplotype diversity; *P*i: nucleotide diversity.

**Table 2:** Results of analysis of molecular variance (AMOVA) of the concatenated cpDNA sequences, chloroplast sequences from northeast and southwest populations of the RRF, as well as nDNA sequences in all populations of *C. dolichophylla*.

Markers	Source of variation	d.f.	Sum of squares	Variance components	Percentage of variation (%)	*F*st	*N*m
cpDNA	Among populations	12	676.16	5.53	83.68	0.84	0.05
	Within populations	117	126.10	1.08	16.32		
Northeast	Among populations	6	242.54	3.91	74.59	0.75	0.08
	Within populations	63	83.90	1.33	25.41		
Southwest	Among populations	5	182.87	3.58	82.08	0.82	0.05
	Within populations	54	42.20	0.78	17.92		
*GTP*	Among populations	12	167.90	0.67	55.08	0.55	0.20
	Within populations	247	135.40	0.55	44.92		
*PHYP*	Among populations	12	213.93	0.85	50.90	0.51	0.24
	Within populations	247	202.65	0.82	49.10		
*PPRC*	Among populations	12	174.91	0.70	59.24	0.59	0.17
	Within populations	247	119.75	0.48	40.76		

D.f.: degrees of freedom; *F*_ST_: the fixation index; *N*m: gene flows.

**Table 3:** Genetic diversity and differentiation parameters (mean ± SE in parentheses) for the jointed cpDNA matrix, chloroplast sequences from northeast and southwest populations of the RRF, as well as nDNA sequences in all populations of *C. dolichophylla.*

Markers	*H*_S_	*H*_T_	*N*_ST_	*G*_ST_	U test
cpDNA	0.320 (0.082)	0.994 (0.014)	0.834 (0.063)	0.678 (0.082)	*N*st > *G*st*significant
Northeast	0.321 (0.121)	0.976 (0.041)	0.740 (0.121)	0.672 (0.125)	non-significant
Southwest	0.319 (0.119)	1.000 (0.036)	0.823 (0.099)	0.681 (0.119)	significant
*GTP*	0.387 (0.061)	0.719 (0.084)	0.551 (0.144)	0.463 (0.121)	non-significant
*PHYP*	0.524 (0.068)	0.869 (0.045)	0.509 (0.118)	0.397 (0.099)	non-significant
*PPRC*	0.291 (0.072)	0.670 (0.106)	0.592 (0.094)	0.565 (0.102)	non-significant

*H*s: the mean genetic diversity within populations; *H*_T_: total genetic diversity for species; *G*_ST_ and *N*_ST_: coefficient of population differentiation.

*: P < 0.05, significant difference.
