# Supplementary material for: Tectonic and climatic impacts on the biota within the Red River Fault, evidence from phylogeography of Cycas dolichophylla (Cycadaceae)
Source: Sci Rep. 2016 Sep 15;6:33540. doi: 10.1038/srep33540 (PMC5024324; doi:10.1038/srep33540)
Supplement: Supplementary Information [file srep33540-s1.docx]

**Title:** Tectonic and climatic impacts on the biota within the Red River Fault, evidence from phylogeography of *Cycas dolichophylla* (Cycadaceae)

**Authors:** Ying Zheng^1,2^, Jian Liu^1,2^, Xun Gong^1*^


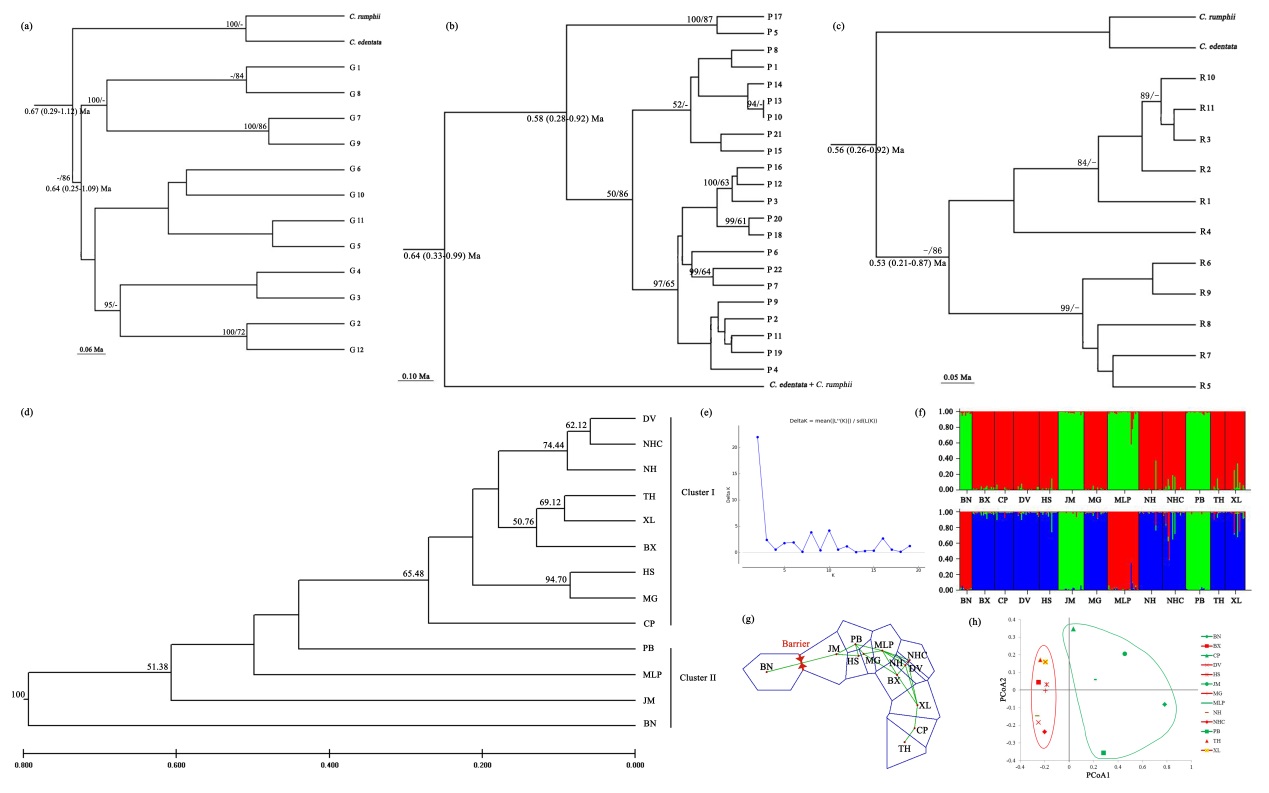


**Figure S1** BEAST-generated maximum clade credibility trees for three nDNAs as well as UPGMA, STRUCTURE, BARRIER and PC_O_ analysis for SSR. (a), (b), (c): The BEAST trees of *GTP*, *PHYP* and *PPRC*, respectively, accorded with Bayesian Inference and Maximum Parsimony, except for minor incongruences at nodes with low confidence level. Numbers above the branches indicate posterior probabilities (left) and bootstrap values (right) from the Maximum Parsimony principle (>50 are shown). Numbers below branches denote the divergence times with 95% HPD. (d): An unweighted pair-group method with arithmetic mean (UPGMA) phenogram of 13 populations of *C. dolichophylla*, based on 16 SSR markers. Numbers on branches indicate bootstrap values from 5000 replicates with values under 50 unshow. (e) and (f): Estimated genetic clustering obtained by STRUCTURE based on sequenced SSR data. (f): Upper: K = 2; lower: K = 3. (g): The boundary between 13 sampled populations of *C. dolichophylla* detected by the BARRIER program based on SSR matrices of unbiased genetic distance. (h): Principal coordinates (PC_O_) analysis of SSR phenotype from 13 populations of 255 individuals of *C. dolichophylla.* Populations scattered in the green circle are from China except for CP and the remaining eight populations restricted to red circle are mainly from Vietnam with HS and MG as exceptions.


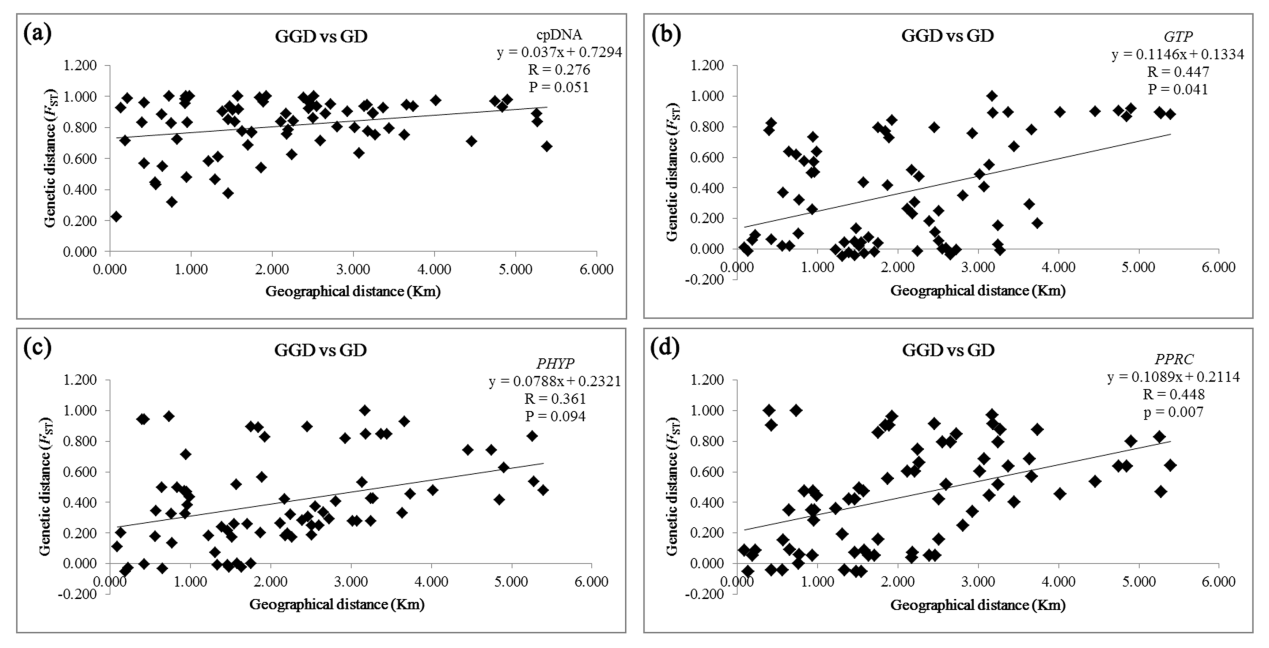


**Figure S2** Analysis of isolation by distance for cpDNA (a) and nDNA (b: *GTP*; c: *PHYP*; d: *PPRC*) sequences in *C. dolichophylla.*


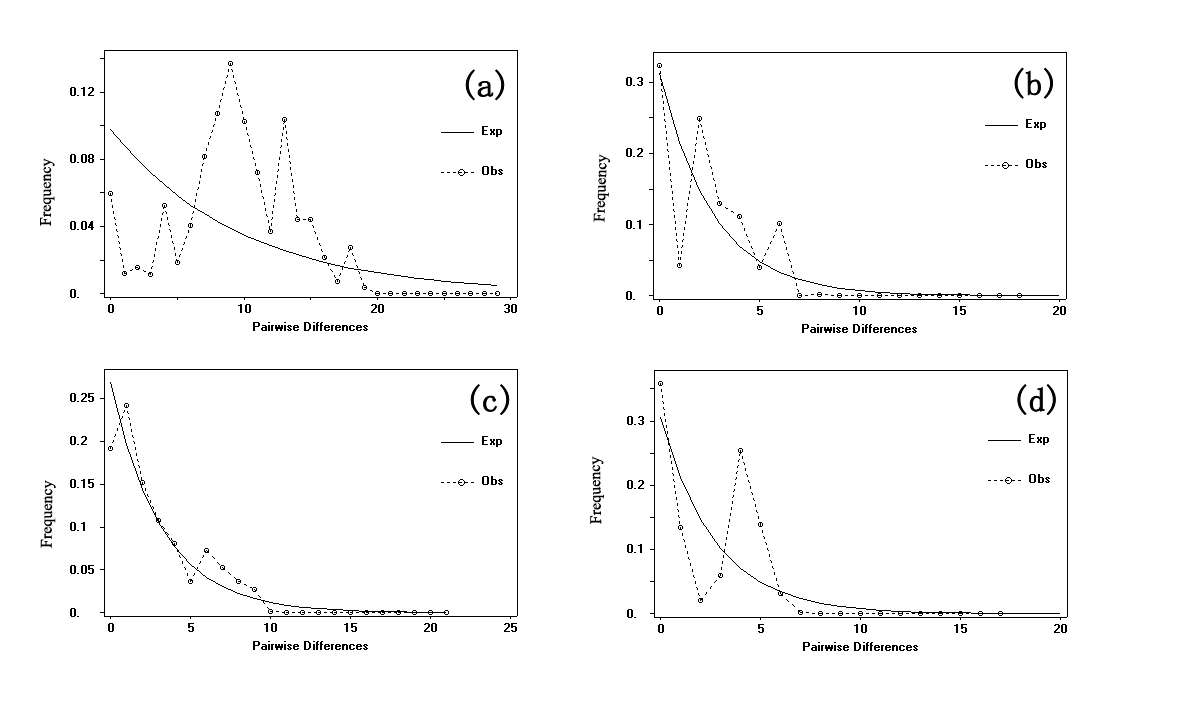


**Figure S3** Mismatch distribution of cpDNA (a) and nDNA (b: *GTP*; c: *PHYP*; d: *PPRC*) haplotypes based on pairwise sequence difference against the frequency of occurrence for *C. dolichophylla* used in this study.

**Table S1** Genetic diversity analysis of the 16 microsatellite loci in *C. dolichophylla*

| **Loci** | ***N*_T_** | ***N*a** | ***N*e** | ***A*_R_** | ***I*** | ***H*o** | ***H*e** | ***UH*e** | ***F*is** | ***F*it** | ***F*st** | ***H*s** | ***H*_T_** | ***D*est** | ***G*st** | ***G*st'** | ***N*m** |
| --- | --- | --- | --- | --- | --- | --- | --- | --- | --- | --- | --- | --- | --- | --- | --- | --- | --- |
| Cha06 | 18 | 4.231 | 2.086 | 3.623 | 0.856 | 0.258 | 0.446 | 0.459 | 0.421 | 0.613 | 0.331 | 0.464 | 0.728 | 0.264 | 0.363 | 0.381 | 0.506 |
| Cha08 | 23 | 7.923 | 4.147 | 6.664 | 1.543 | 0.698 | 0.679 | 0.699 | -0.027 | 0.202 | 0.223 | 0.698 | 0.873 | 0.175 | 0.200 | 0.213 | 0.872 |
| cha-est01 | 47 | 10.538 | 6.220 | 8.574 | 1.929 | 0.405 | 0.786 | 0.808 | 0.485 | 0.566 | 0.157 | 0.819 | 0.952 | 0.133 | 0.139 | 0.149 | 1.340 |
| cha-est02 | 8 | 3.231 | 2.059 | 3.011 | 0.781 | 0.240 | 0.442 | 0.454 | 0.458 | 0.623 | 0.304 | 0.46 | 0.623 | 0.163 | 0.261 | 0.277 | 0.572 |
| cha-est03 | 7 | 1.385 | 1.088 | 1.319 | 0.106 | 0.076 | 0.061 | 0.063 | -0.252 | 0.616 | 0.694 | 0.062 | 0.289 | 0.227 | 0.785 | 0.798 | 0.110 |
| cha-est05 | 10 | 3.615 | 2.049 | 3.266 | 0.820 | 0.396 | 0.447 | 0.460 | 0.115 | 0.445 | 0.373 | 0.461 | 0.716 | 0.255 | 0.356 | 0.374 | 0.420 |
| Cpz8 | 4 | 1.538 | 1.246 | 1.499 | 0.184 | 0.112 | 0.101 | 0.105 | -0.103 | 0.497 | 0.544 | 0.104 | 0.344 | 0.240 | 0.698 | 0.715 | 0.210 |
| Cpz22 | 8 | 2.846 | 1.394 | 2.488 | 0.443 | 0.226 | 0.234 | 0.240 | 0.033 | 0.243 | 0.217 | 0.240 | 0.401 | 0.160 | 0.400 | 0.420 | 0.903 |
| CPZ26 | 20 | 8.308 | 5.274 | 7.070 | 1.726 | 0.699 | 0.756 | 0.777 | 0.076 | 0.227 | 0.164 | 0.779 | 0.91 | 0.130 | 0.143 | 0.154 | 1.275 |
| CY232 | 7 | 3.615 | 2.094 | 3.111 | 0.820 | 0.461 | 0.460 | 0.473 | -0.003 | 0.309 | 0.311 | 0.473 | 0.689 | 0.216 | 0.313 | 0.331 | 0.553 |
| CY270 | 8 | 3.231 | 2.071 | 3.010 | 0.783 | 0.425 | 0.435 | 0.447 | 0.023 | 0.323 | 0.307 | 0.448 | 0.673 | 0.225 | 0.334 | 0.352 | 0.564 |
| Cy-Tai-EST08 | 20 | 6.538 | 4.336 | 5.812 | 1.495 | 0.555 | 0.699 | 0.718 | 0.206 | 0.380 | 0.218 | 0.723 | 0.913 | 0.190 | 0.208 | 0.221 | 0.895 |
| E001 | 13 | 5.385 | 3.207 | 4.748 | 1.281 | 0.612 | 0.643 | 0.661 | 0.049 | 0.193 | 0.151 | 0.662 | 0.780 | 0.118 | 0.151 | 0.162 | 1.402 |
| G46 | 3 | 1.923 | 1.204 | 1.743 | 0.265 | 0.147 | 0.151 | 0.156 | 0.029 | 0.100 | 0.073 | 0.156 | 0.163 | 0.008 | 0.047 | 0.051 | 3.189 |
| HL01 | 7 | 3.385 | 1.934 | 3.038 | 0.774 | 0.349 | 0.445 | 0.457 | 0.215 | 0.461 | 0.314 | 0.461 | 0.660 | 0.200 | 0.303 | 0.320 | 0.547 |
| HL08 | 14 | 6.154 | 3.473 | 5.390 | 1.384 | 0.732 | 0.666 | 0.684 | -0.099 | 0.112 | 0.192 | 0.683 | 0.829 | 0.147 | 0.177 | 0.189 | 1.050 |
| Mean | 13.563 | 4.615 | 2.743 | 4.023 | 0.949 | 0.399 | 0.466 | 0.479 | 0.102 | 0.369 | 0.286 | 0.481 | 0.659 | 0.178 | 0.270 | 0.286 | 0.901 |

*N*_T_: total number of alleles; *N*a: the mean number of alleles; *N*e: effective number of alleles; *A*_R_: Allelic richness; *I*: Shannon’s diversity index; *H*_O_: the observed heterozygosity; *H*e: the expected heterozygote; *UH*e: Nei’s unbiased heterozygosity; *F*is*, F*it and *F*st: the fixation index; *H*s: the average within-population diversity; *H*_T_: the total diversity; *D*est, *G*st and *G*st’: coefficience of gene differentiations; *N*m: gene flows. The same codes for the following parametres.

**Table S2** Genetic variability of the 16 microsatellites within 13 *C. dolichophylla* populations

| **Populations** | ***N*_T_** | ***N*a** | ***N*e** | ***N*p** | ***A*_R_** | ***H*o** | ***H*e** | ***UH*e** | ***I*** | ***F*** | ***PPB*(%)** |
| --- | --- | --- | --- | --- | --- | --- | --- | --- | --- | --- | --- |
| BN | 65 | 4.063 | 2.630 | 11 | 4.063 | 0.517 | 0.476 | 0.499 | 0.938 | -0.108 | 87.50 |
| BX | 94 | 5.875 | 3.460 | 20 | 4.864 | 0.459 | 0.508 | 0.521 | 1.122 | 0.076 | 87.50 |
| CP | 50 | 3.125 | 2.193 | 17 | 2.879 | 0.364 | 0.410 | 0.422 | 0.734 | 0.168 | 87.50 |
| DV | 86 | 5.375 | 2.872 | 23 | 4.385 | 0.391 | 0.474 | 0.484 | 1.022 | 0.187 | 87.50 |
| HS | 75 | 4.688 | 2.870 | 17 | 4.200 | 0.371 | 0.471 | 0.486 | 0.974 | 0.166 | 87.50 |
| JM | 61 | 3.813 | 2.191 | 23 | 3.337 | 0.348 | 0.417 | 0.426 | 0.801 | 0.108 | 93.75 |
| MG | 72 | 4.500 | 2.490 | 21 | 3.783 | 0.372 | 0.465 | 0.477 | 0.925 | 0.223 | 87.50 |
| MLP | 69 | 4.313 | 2.345 | 28 | 3.426 | 0.350 | 0.421 | 0.428 | 0.828 | 0.141 | 81.25 |
| NH | 79 | 4.938 | 2.749 | 21 | 4.173 | 0.393 | 0.478 | 0.489 | 0.994 | 0.171 | 87.50 |
| NHC | 69 | 4.313 | 2.753 | 21 | 3.835 | 0.405 | 0.484 | 0.496 | 0.967 | 0.146 | 93.75 |
| PB | 42 | 2.625 | 1.748 | 22 | 2.328 | 0.281 | 0.327 | 0.334 | 0.554 | 0.115 | 75.00 |
| TH | 95 | 5.938 | 3.471 | 13 | 5.545 | 0.471 | 0.540 | 0.561 | 1.195 | 0.111 | 100.00 |
| XL | 103 | 6.438 | 3.881 | 18 | 5.482 | 0.469 | 0.584 | 0.601 | 1.289 | 0.181 | 87.50 |
| Mean | 73.846 | 4.615 | 2.743 | 19.615 | 6.290 | 0.399 | 0.466 | 0.479 | 0.949 | 0.129 | 87.98 |

*N*p: the number of private alleles; *PPB*: the percentage of polymorphic loci.

**Table S3** Gene flows between each pair of the 13 populations in *C. dolichophylla* based on SSR data

| **Populations** | **BN** | **BX** | **CP** | **DV** | **HS** | **JM** | **MG** | **MLP** | **NH** | **NHC** | **PB** | **TH** | **XL** |
| --- | --- | --- | --- | --- | --- | --- | --- | --- | --- | --- | --- | --- | --- |
| BN | 0.000 |  |  |  |  |  |  |  |  |  |  |  |  |
| BX | 0.786 | 0.000 |  |  |  |  |  |  |  |  |  |  |  |
| CP | 0.623 | 2.128 | 0.000 |  |  |  |  |  |  |  |  |  |  |
| DV | 0.906 | 4.736 | 1.473 | 0.000 |  |  |  |  |  |  |  |  |  |
| HS | 0.858 | 2.484 | 1.827 | 2.308 | 0.000 |  |  |  |  |  |  |  |  |
| JM | 0.586 | 1.120 | 0.889 | 0.884 | 0.877 | 0.000 |  |  |  |  |  |  |  |
| MG | 0.921 | 2.052 | 1.933 | 1.961 | 6.329 | 0.900 | 0.000 |  |  |  |  |  |  |
| MLP | 0.852 | 1.108 | 1.020 | 1.218 | 0.993 | 0.596 | 0.995 | 0.000 |  |  |  |  |  |
| NH | 0.806 | 4.785 | 1.878 | 8.119 | 2.455 | 0.982 | 2.276 | 1.159 | 0.000 |  |  |  |  |
| NHC | 1.105 | 3.092 | 1.545 | 7.909 | 2.828 | 0.955 | 2.719 | 1.339 | 4.488 | 0.000 |  |  |  |
| PB | 0.553 | 1.145 | 0.721 | 1.406 | 0.999 | 0.670 | 0.944 | 0.623 | 1.318 | 1.602 | 0.000 |  |  |
| TH | 0.970 | 4.749 | 2.630 | 3.148 | 4.720 | 1.577 | 3.416 | 1.212 | 3.689 | 2.920 | 1.058 | 0.000 |  |
| XL | 1.010 | 4.071 | 2.466 | 2.823 | 3.428 | 1.450 | 2.988 | 1.325 | 3.128 | 3.441 | 1.105 | 6.600 | 0.000 |

**Table S4** Parameters of neutrality tests and demographic analysis based on cpDNA sequences as a whole, cpDNA populations from southwestern and northeastern of RRF and three nDNA sequences of *C. dolichophylla.*

| **Marker** | **Tajima’s D** | **Fu and Li’s D*** | **Fu and Li’s F*** | **Fu’s Fs** | **SSD** | **raggedness** |
| --- | --- | --- | --- | --- | --- | --- |
| cpDNA | -0.46999 | 1.04439 | 0.48054 | 1.195 | 0.08018* | 0.36748 |
| Northeast | -0.19761 | 0.49569 | 0.27824 | 3.038 | 0.05769 | 0.34977 |
| Southwest | 0.31924 | 0.53320 | 0.54297 | 3.602 | 0.10642 | 0.38815 |
| *GTP* | -0.08783 | 1.51131 | 1.08280 | 0.237 | 0.10921 | 0.41809 |
| *PHYP* | -0.42467 | 0.14475 | -0.10034 | -1.890 | 0.05108 | 0.19133 |
| *PPRC* | 0.62348 | 1.36604 | 1.30965 | 0.394 | 0.05456 | 0.36456 |

*is P < 0.05, significant difference

**Table S5** Bottleneck analysis for 13 populations of *C. dolichophylla* based on SSR data

| **Populations** | **T.P.M** | | **S.M.M** | | **Mode shift** | **G-W** |
| --- | --- | --- | --- | --- | --- | --- |
|  | **Sign test** | **Wilcoxon test** | **Sign test** | **Wilcoxon test** |  |  |
| BN | 0.05755 | 0.04431* | 0.48523 | 0.32251 | L | 0.430 |
| BX | 0.28982 | 0.25223 | 0.34746 | 0.89993 | L | 0.420 |
| CP | 0.11969 | 0.01550* | 0.55063 | 0.29785 | L | 0.286 |
| DV | 0.2685 | 0.19281 | 0.17878 | 0.41045 | L | 0.387 |
| HS | 0.26999 | 0.08325 | 0.45669 | 0.63217 | L | 0.411 |
| JM | 0.28447 | 0.05066 | 0.48258 | 0.49542 | L | 0.443 |
| MG | 0.27659 | 0.17535 | 0.17598 | 0.66855 | L | 0.405 |
| MLP | 0.43286 | 0.78195 | 0.36808 | 0.66855 | L | 0.393 |
| NH | 0.45158 | 0.97995 | 0.54862 | 0.52817 | L | 0.342 |
| NHC | 0.05299 | 0.00919** | 0.29248 | 0.95837 | L | 0.429 |
| PB | 0.03656* | 0.00168** | 0.11338 | 0.05066 | L | 0.385 |
| TH | 0.17269 | 0.27444 | 0.15688 | 0.56189 | L | 0.372 |
| XL | 0.28866 | 0.09344 | 0.31650 | 0.63217 | L | 0.352 |

*: P < 0.05, significant difference; **: P < 0.01, most significant difference.

**Table S6** Effective population sizes (*N*_E_) derived from LDNe on the base of SSR data of *C. dolichophylla*

| **Populations** | **BN** | **BX** | **CP** | **DV** | **HS** | **JM** | **MG** | **MLP** | **NH** | **NHC** | **PB** | **TH** | **XL** | **Mean** |
| --- | --- | --- | --- | --- | --- | --- | --- | --- | --- | --- | --- | --- | --- | --- |
| *N*_E_ | 7.9 | 96.2 | 0.7 | 37.9 | 15.8 | 30 | 9.7 | 69.2 | 23.3 | 9.1 | -45.9 | -43.5 | 19 | 17.6 |

**Table S7** Details of sampling information of the 13 populations of *C. dolichophylla* along the RRF.

| **Code** | **Location** | **Latitude (°N)** | **Longitude (°E)** | **Altitude (m)** | **Number of samples** |
| --- | --- | --- | --- | --- | --- |
| BN | Nabanhe Nature Reserve, Yunnan, China | 22.1277 | 100.6771 | 672 | 11 |
| BX | Binh Xa, Vietnam | 22.0338 | 105.1365 | 118 | 20 |
| CP | Cuc Phuong, Vietnam | 20.2621 | 105.7372 | 301 | 17 |
| DV | Davi Commune, Vietnam | 22.4559 | 105.5109 | 173 | 23 |
| HS | Hekou County, Yunnan, China | 22.6653 | 103.8186 | 573 | 17 |
| JM | Jinping County, Yunnan, China | 22.7219 | 103.0613 | 960 | 23 |
| MG | Maguan County, Yunnan, China | 22.7317 | 103.9949 | 604 | 21 |
| MLP | Malipo County, Yunnan, China | 22.8419 | 104.6322 | 577 | 28 |
| NH | Na Hang, Vietnam | 22.3539 | 105.4228 | 230 | 21 |
| NHC | Na Hang, Vietnam | 22.5228 | 105.5663 | 851 | 21 |
| PB | Pingbian County, Yunnan, China | 23.0534 | 103.7137 | 731 | 22 |
| TH | Thanh Hoa, Vietnam | 19.7999 | 105.3969 | 110 | 13 |
| XL | Xuan Lien, Vietnam | 21.0281 | 105.8343 | 405 | 18 |
| Total |  |  |  |  | 255 |
